# Sexually antagonistic selection maintains genetic variance when sexual dimorphism evolves

**DOI:** 10.1098/rspb.2022.2484

**Published:** 2023-03-29

**Authors:** Philipp Kaufmann, James Malcolm Howie, Elina Immonen

**Affiliations:** ^1^ Department of Ecology and Genetics (Evolutionary Biology program), Uppsala University, Norbyvägen 18D, 75234 Uppsala, Sweden; ^2^ Institute of Forest Entomology, Forest Pathology and Forest Protection, Boku, University of Natural Resources and Life Sciences, Peter-Jordan-Straße 82/I, 1190, Vienna, Austria

**Keywords:** sexual conflict, balancing selection, body size, dominance variation, artificial selection, animal model

## Abstract

Genetic variance (*V*_G_) in fitness related traits is often unexpectedly high, evoking the question how *V*_G_ can be maintained in the face of selection. Sexually antagonistic (SA) selection favouring alternative alleles in the sexes is common and predicted to maintain *V*_G_, while directional selection should erode it. Both SA and sex-limited directional selection can lead to sex-specific adaptations but how each affect *V*_G_ when sexual dimorphism evolves remain experimentally untested. Using replicated artificial selection on the seed beetle *Callosobruchus maculatus* body size we recently demonstrated an increase in size dimorphism under SA and male-limited (ML) selection by 50% and 32%, respectively. Here we test their consequences on genetic variation*.* We show that SA selection maintained significantly more ancestral, autosomal additive genetic variance than ML selection, while both eroded sex-linked additive variation equally. Ancestral female-specific dominance variance was completely lost under ML, while SA selection consistently sustained it. Further, both forms of selection preserved a high genetic correlation between the sexes (*r*_m,f_). These results demonstrate the potential for sexual antagonism to maintain more genetic variance while fuelling sex-specific adaptation in a short evolutionary time scale, and are in line with predicted importance of sex-specific dominance reducing sexual conflict over alternative alleles.

## 1. Introduction

Natural selection acts on the heritable genetic variance in fitness-related traits, yet the additive genetic variance (*V*_A_) for such traits is often unexpectedly high [1–6]. Besides the mutation-selection balance [7,8], an answer to how genetic variation can be maintained should lie in the strength, direction and type of selection acting on a trait. Variable selection due to biotic and abiotic environment, population structure, genotype or sex, can all influence selection and maintain genetic variance when different alleles are favoured in different individuals or at different time points [9]. For traits subject to sexually antagonistic (SA) selection, alleles are selected for or against depending on the sex they reside in. SA selection is common in nature [10] and stems from discordant reproductive strategies in females and males [11–13]. SA selection is predicted to maintain genetic variation through a net balancing selection across the sexes [14–17]. Observed signatures of balancing selection at the genomic level—such as elevated nucleotide diversity in proximity to sexually antagonistic genes—are compatible with this prediction [18]. Empirical evidence is currently sparse and limited to molecular studies where SA selection is approximated indirectly from inferences of sex-biased gene expression [18,19] or where the estimates of SA selection and balanced polymorphism are obtained from different populations [20]. What is especially warranted is a direct test that allows causally connecting the effects of SA selection on the level of maintenance of genetic variance in a trait subject to antagonistic selection in the sexes. The theoretical framework is based on a single locus scenario, and its applicability to polygenic traits remains to be tested.

How selection affects genetic variance across the sexes depends on the degree to which shared genetic variation affects joint phenotypic variation in the two sexes, which is captured by the intersexual genetic correlation (*r*_m,f_). A positive *r*_m,f_ sets the stage for intralocus sexual conflict when the sexes have different fitness optima [21,22] and can impede independent trait evolution in the sexes [23] but see [24,25]. Following this argument, a reduction of *r*_m,f_ has been considered as a potential prerequisite to the evolution of sexual dimorphism [26–28]. Consistent with this, studies have found a negative correlation between the degree of dimorphism and *r*_m,f_ when compared across different traits [29–31]. However, the evidence is equivocal [22,32] and the consequence of SA selection [33], or other forms of sex-specific selection, on the *r*_m,f_ during the evolution of sexual dimorphism is not well understood. Studies comparing the *r*_m,f_ across different types of traits [30] or genes [31] focus on longer evolutionary time scales, but empirical studies investigating shorter time scales relevant for the evolution of sex-specific adaptations from standing genetic variation (from which the *r*_m,f_ is estimated) are lacking.

While theory suggests that SA selection can maintain shared genetic variance, and therefore also the *r*_m,f_, SA selection by itself is not sufficient [34]: maintenance of genetic variance through SA selection is restricted to a narrow parameter space under additivity and sex-concordant dominance [15,17,35]. Sex-specific differences in dominance and particularly sex-specific dominance reversal greatly expand this parameter space and make maintenance of genetic variance a likely outcome of SA selection [17,36]. However, it is not well understood how often these dominance scenarios are met and the role of sex-specific dominance in evolution has been controversial [6,36–39]. Sex-specific dominance modifiers may evolve in response to SA selection [16] and facilitate partial resolution of sexual conflict if alleles favoured in a given sex are dominant over the alleles selected against in that sex [36,40]. SA selection could also maintain genetic variance through the effect of dominance that emerges from diminishing returns of allelic contribution to fitness (or polygenic traits closely related to fitness), i.e. the concavity of the fitness surface within each sex, even without evoking dominance allelic effects for such traits *per se* (e.g. [14,36,39]). Examples of sex-specific dominance in major-effect loci across multiple species (e.g. [41–43]) showcase that sex-specific dominance does occur and suggests that genetic variance for such loci can persist across different selection contexts. Polygenic dominance variance reversal for fitness in *C. maculatus* [4] highlights the potential for SA to maintain genetic variance genome wide.

Here we empirically quantify how additive and dominance genetic variances, and the *r*_m,f_, are affected by evolution of sexual body size dimorphism (SSD) by artificial selection in a seed beetle *Callosobruchus maculatus,* and specifically test whether SA selection can maintain genetic variation at a higher level compared to sex-limited directional selection. Body size is commonly sexually dimorphic, and especially interesting trait given its strong association with life-history variation, ecological adaptation and speciation rates across taxa [44,45]. Fitness optima for body size are different for the sexes in *C. maculatus* population used for this study [46–48], and in the context of the artificial selection regimes body weight solely determined fitness. This study builds upon our recent work [24] where we described the genetic variances associated with *C. maculatus* body size (electronic supplementary material, table S1) in a wild-type ancestral population and experimentally demonstrated that sexual size dimorphism increased under both SA selection (toward smaller males and larger females) and male-limited (ML) selection (toward smaller males), by 50% and 32%, respectively. These selection forms affected male size similarly (the cumulative selection differential of male size and consequently the phenotypic response were similar in males (electronic supplementary material, figures S1 and S2)), while the SA selection prevented a correlational change in female size seen under ML selection, resulting in the greater increase in dimorphism under SA selection. We showed that the autosomal additive genetic variance was highly correlated between the sexes in the ancestral population ($r_{m,f}^{a}\;=\;0.924$). The rapid evolution of SSD could largely be explained by sex-limited genetic variances: the X- and particularly Y-linked additive genetic variance associated with male size and the dominance variance associated with female body size [24]).

Answering the question how short-term selection affects standing genetic variance for a polygenic trait such as the body size depends on numerous assumptions, and is ultimately an empirical question yet to be answered (see electronic supplementary material, Results). Nonetheless, predicting broadly, sex-limited directional selection is expected to erode additive genetic variance more quickly than SA selection. This is because sex-limited selection can cause an unhindered correlated response in the opposite sex [24], driving allele frequencies underlying sex-specific but also sex-shared additive genetic variation towards extreme values or fixation, reducing or completely eroding genetic variances in the sexes. SA selection can similarly erode sex-specific additive variance but could maintain shared additive genetic variance by favouring alternative alleles in the two sexes [17]. Note that sex-shared variance (covariance) and sex-limited variance may also not respond to selection independently but could be interconnected by pleiotropy or linkage during selection.

Predicting the consequences of selection on the detected female-limited dominance variance requires also considering its effects on the opposite sex. Male limited-selection is completely blind to the effects of female dominance variance. If, on the one hand, the loci underlying female dominance variation have additive effects on males, ML selection is expected to push allele frequencies toward extreme values or fixation. If, on the other hand, the underlying loci have no effect on the male phenotype, selection on males only will leave such loci susceptible to drift. Consequently, female-specific dominance variance is expected to be reduced or lost under ML selection. By contrast, because heterozygosity is necessary for dominance effects to manifest themselves, and if sexual dimorphism is facilitated by the detected female-specific dominance, then SA selection should favour the expression of dominance effects by driving underlying allele frequencies to intermediate frequencies, where dominance variance is highest [49]. If the loci underlying female dominance have instead no effect on the male phenotype (and are hence not sexually antagonistic), then dominance variance is expected to be reduced or lost in the SA selection regime as well.

Thus, unlike sex-limited directional selection, SA selection for increased sexual dimorphism could maintain or even increase dominance variance, in addition to maintaining the additive genetic variance. No change in the *r*_m,f_ is necessarily predicted under either selection regime, if SA maintains and ML selection reduces the *V*_A_ equally in the sexes, but both forms of selection are expected to erode the sex-linked additive genetic variances. We test these predictions by comparing the variances between the replicated SA and ML lines, to the ancestral levels, as well as to the effects produced by genetic drift alone.

## 2. Results

### (a) Sexually antagonistic selection maintains additive and dominance variance

We conducted repeated variance decomposition analysis using Bayesian mixed effects models before and after selection using two independent replicate lines subjected to sexually antagonistic (SA), male-limited (ML) or random selection (RS) (each of the six selection line pedigrees contains approximately 2000 individuals, so combined with the ancestral pedigree our dataset includes 22 000 individuals). First, we found that a significant amount of autosomal additive genetic variance remained in both sexes after selection ($V_{A,f}^{a}$ & $V_{A,m}^{a}$ for females and males, respectively) for all lines, although the extent was reduced compared to the ancestral population in all but one line (RS_b_) (electronic supplementary material, tables S1–S4). By contrast, the sex-linked additive variance components observed in males of the ancestral population were not maintained: Y-linked variance ($V_{A}^{Y}$) for male body size [24] was significantly reduced in all, and lost completely in most lines (electronic supplementary material, figures S3*f*,*h*, S4*f*,*h* and S5*f*,*h*), and male X-linked genetic variance ($V_{A,m}^{X}$) was lost in all lines (electronic supplementary material, tables S1–S4). No X-linked additive genetic variance was detected in females ($V_{A,f}^{X}$) either before or after selection in any of the lines.

**Figure 1.**
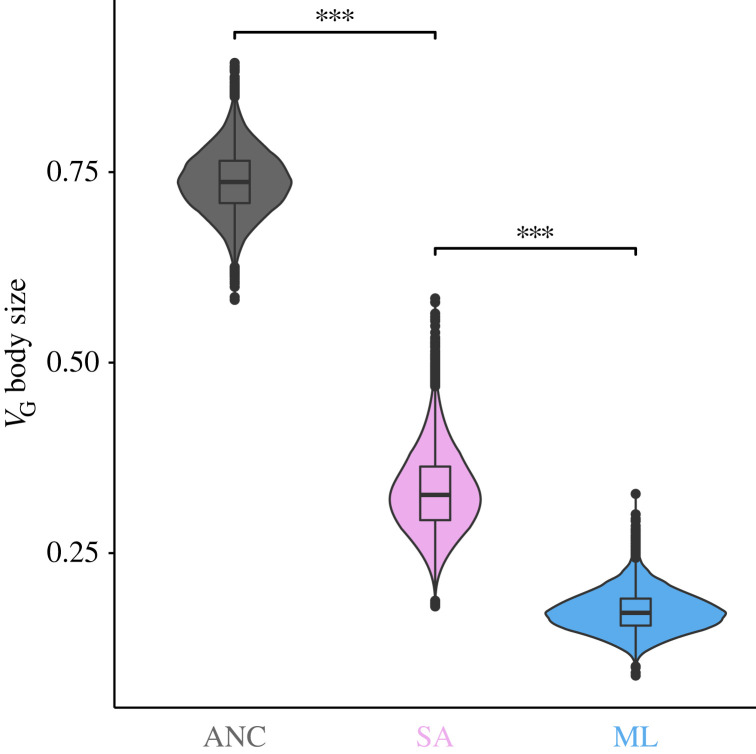
SA selection maintained more total genetic variance (*V*_G_) for body size (across the sexes) relative to the directional selection limited to males. Violin plots show the posterior distribution of *V*_G_ in the ancestral (ANC) population and after ten generations of artificial male-limited (ML) and sexually antagonistic (SA) selection. *** = posterior difference 99.99% CI does not overlap zero. Note that replicate lines a and b are presented together because they do not significantly differ among the replicate lines within selection regimes, replicate specific comparisons are presented in electronic supplementary material, figure S6.

**Figure 2.**
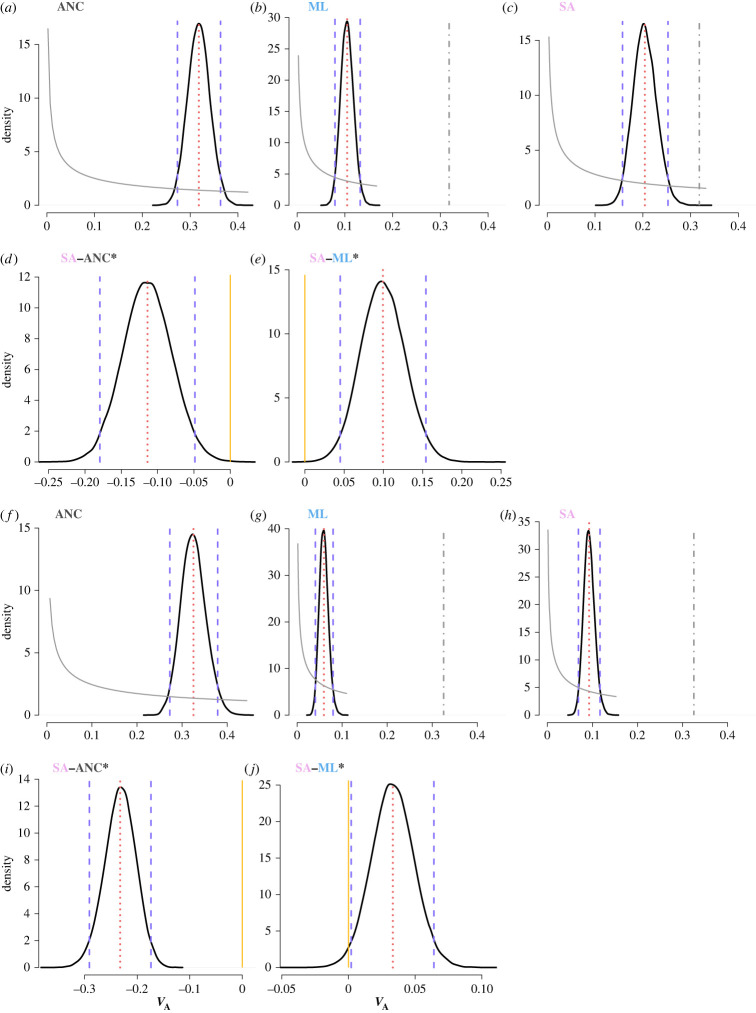
More additive genetic variance (*V*_A_) is maintained under SA selection in (*a–e*) females and (*f–j*) males. Panels (*a*,*f*) show *V*_A_ in the ancestral (ANC) population, while (*b*,*g*) show the evolved patterns after ten generations of artificial male-limited (ML) and (*c*,*h*) of sexually antagonistic (SA) selection. Panels (*d*,*i*) and (*e*,*j*) show the posterior difference between SA-ANC and SA-ML. Indicated are the marginal posterior distributions (solid black lines), 95% credible intervals (vertical dashed purple lines), posterior means (vertical dotted red lines) and prior distributions (solid grey lines). In panels (*b*,*c*) we additionally show the mean variance of the ancestral population (vertical dot-dashed grey line) to guide comparison. Asterisks indicate posterior difference distributions significantly greater than zero, i.e. where the 95% credible interval does not overlap 0 (highlighted with a vertical yellow line). Note the different scales on the *x*-axis and *y*-axis between different plots.

**Figure 3.**
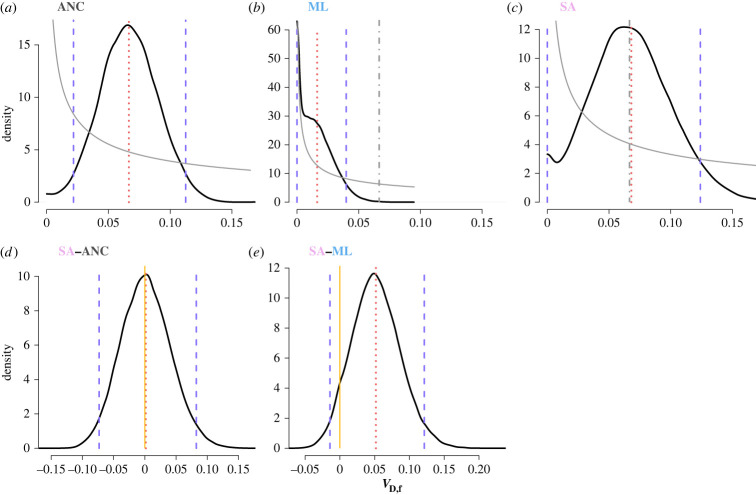
SA selection maintained more female dominance variance (V_D_) for body size relative to the directional selection limited to males. Marginal posterior distributions of female dominance variance in (*a*) the ancestral (ANC) population and in the populations after ten generations of (*b*) artificial male-limited (ML) and (*c*) sexually antagonistic (SA) selection. Panels (*d*) and (*e*) show the posterior difference between SA- ANC and SA-ML, respectively. Indicated are the marginal posterior distributions (solid black lines), 95% credible intervals (vertical dashed purple lines), posterior means (vertical dotted red lines) and prior distributions (solid grey lines). In panels (*b*,*c*) we additionally show the mean variance of the ancestral population (vertical dot-dashed grey line) to guide comparison. Asterisks indicate posterior difference distributions significantly greater than zero, i.e. where the 95% credible interval does not overlap 0 (highlighted with a vertical yellow line). Note the different scales on the *x*-axis and *y*-axis between different plots.

Importantly, despite an overall reduction of genetic variance (*V*_G_) in the lines, SA selection preserved significantly more genetic variance across the sexes and replicate lines than ML selection (figure 1) (*V*_G_ posterior difference 95% CI = 0.096,0.279, measured as the difference between the posterior distribution in the SA regime minus the posterior distribution of the ML regime, electronic supplementary material, figure S6). The pattern holds true whether analysing the replicate lines together or separately (electronic supplementary material, figure S7) or when analysed separately for each sex, SA selection maintained significantly more genetic variance in both females (*V*_G,f_ posterior difference 95% CI = 0.071,0.234; figure 2*a–e*) and males (*V*_G,m_ posterior difference 95% CI = 0.001,0.065; figure 2*f–j*) relative to ML selection. When partitioning *V*_G_ to its additive and dominance components we also found that, in line with our main prediction, SA selection maintained significantly more additive genetic variance in both sexes when compared to ML selection (*V*_A,f_ posterior difference 95% CI = 0.045,0.154; *V*_A,m_ posterior difference 95% CI = 0.002,0.064; figure 2). Albeit statistically significant only in the SAb replicate, we also find that SA consistently maintained more between sex covariance compared to ML regime (table 1). SA selection also maintained the ancestral levels of female-specific dominance variance, while under ML selection no signal for dominance variance was retained in either replicate line. The mean difference between female dominance variance in SA and ML selection was 0.052 with approximately 93% of the posterior distribution of the difference greater than 0.001 (figure 3; electronic supplementary material, figures S3 and S4; this result is further supported by a significant likelihood-ratio test using an independent likelihood-based model; see electronic supplementary material, table S6). In fact, female dominance variance was the only variance component that was not significantly reduced by SA selection relative to the ancestor (mean *V*_D,f_ posterior difference = 0.002, 95% CI = −0.073,0.082; figure 3). We could not detect any dominance variance for male body size either before or after selection (electronic supplementary material, tables S1–S4).

**Table 1.** Posterior estimates of the intersexual covariance (${COV}_{A}^{a}$) for each selection line on the diagonal, as well as the posterior difference in the covariance between each selection line below the diagonal. Shown are the [posterior mode] and (95% credible interval). Significant posterior differences (i.e. 95% credible interval does not overlap zero) are highlighted in bold.

	SAa	SAb	MLa	MLb
SAa	${\mathrm{COV}}_{A}^{a}\mathrm{SAa}$			
	[697.4]			
	(412.6,1046.4)			
Sab	${\mathrm{COV}}_{A}^{a}\mathrm{SAa}-{\mathrm{COV}}_{A}^{a}\mathrm{SAb}$	${\mathrm{COV}}_{A}^{a}\mathrm{SAb}$		
	[−330.2]	[1084.3]		
	(−839.3,86.3)	(776.6,1448.6)		
MLa	${\mathrm{COV}}_{A}^{a}\mathrm{SAa}-{\mathrm{COV}}_{A}^{a}\mathrm{MLa}$	${\text{C}OV}_{\text{A}}^{\text{a}}\text{S}Ab-{\text{C}OV}_{\text{A}}^{\text{a}}\text{M}La$	${\mathrm{COV}}_{A}^{a}\mathrm{MLa}$	
	[289.9]	**[627.8]**	[457.8]	
	(−122.78,639.2)	**(237.4,1027.8)**	(267.1,678.6)	
MLb	${\mathrm{COV}}_{A}^{a}\mathrm{SAa}-{\mathrm{COV}}_{A}^{a}\mathrm{MLb}$	${\text{C}OV}_{\text{A}}^{\text{a}}\text{S}Ab-{\text{C}OV}_{\text{A}}^{\text{a}}\text{M}Lb$	${\mathrm{COV}}_{A}^{a}\mathrm{MLa}-{\mathrm{COV}}_{A}^{a}\mathrm{MLb}$	${\mathrm{COV}}_{A}^{a}\mathrm{MLb}$
	[108.0]	**[465.9]**	[−148.3]	[624.7]
	(−300.5,483.4)	**(73.8,885.6)**	(−476.6,139.4)	(420.7/873.4)

Standing genetic variance estimates after 10 generations of selection were more consistent across the experimental replicates of the sex-specific selection regimes than under random selection, as we observed significant differences only between the two replicate lines of the random selection regime (RS_a_ and RS_b_), for both autosomal additive variance and female dominance variance (electronic supplementary material, table S3). Comparisons of genetic variances between the SA and ML regimes to each random selection replicate are provided in the electronic supplementary material.

### (b) Evolution of sexual size dimorphism did not alter the intersexual genetic correlation

**Figure 4.**
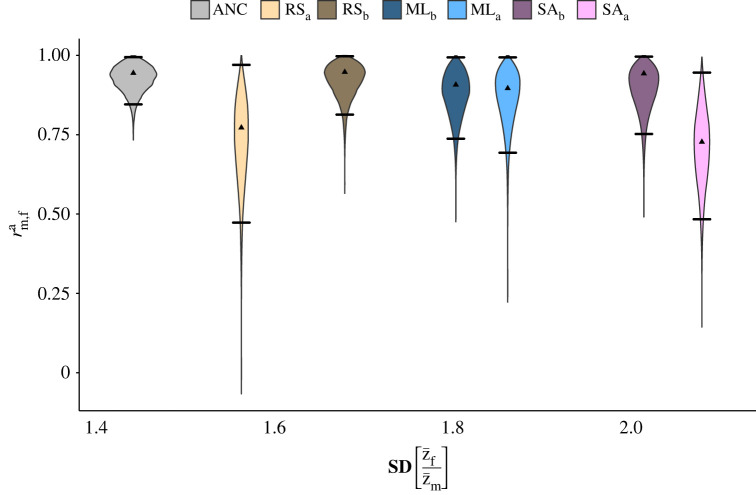
Posterior distributions of the autosomal intersexual genetic correlation plotted against the level of observed sexual dimorphism before (ANC) and after selection (RS, SA, ML). Indicated are the posterior mode (black triangle) and the 95% credible interval (horizontal, black lines).

In our previous study [24] we showed that SA as well as ML selection significantly increased SSD by 50% and 32%, respectively, despite the potential constraint on the evolution of SSD posed by a high intersexual autosomal genetic correlation in the ancestral population ($r_{m,f}^{a}=0.925$). Here we tested whether this evolution of SSD has changed the $r_{m,f}^{a}$. We could not detect any significant reduction in $r_{m,f}^{a}$ between any of the selection lines and the ancestral population, measured as the posterior difference between the $r_{m,f}^{a}$ distributions (electronic supplementary material, table S5). Furthermore, we observed no correlation between the degree of SSD and $r_{m,f}^{a}$ (*F*_1,5_ = 0.5492, *p* = 0.49; figure 4), despite variation in both.

The intersexual genetic correlation is often calculated from quantitative genetic analysis without sex-linked and dominance variance partitioning which can bias the genetic correlation estimate [24,50,51] (hereafter indicated with an asterisk, $r_{m,f}^{*})$. When we calculated $r_{m,f}^{*}$ from our data, we observed a mean increase in $r_{m,f}^{*}$ after selection across all selection lines relative to the ancestor, significantly so in the lines SA_b_ and ML_b_ (electronic supplementary material, figure S8, table S5).

## 3. Discussion

Here we demonstrate that, compared to male-limited selection, SA selection can maintain higher levels of both autosomal additive and dominance genetic variance during short-term evolution of sexual dimorphism. By combining direct estimation of genetic variance before and after body size evolution under SA and ML selection, our study allows us to draw a causal connection between the nature of selection, the type of phenotypic evolution, and their consequences for genetic variances, while controlling for demography. Our previous study, where the selection lines were established, showed that sexual size dimorphism increased by approximately 20% more under SA than ML selection owing to differences in female response [24]. The cumulative strength of selection and consequently the phenotypic response was similar in males under both regimes (electronic supplementary material, figures S1 and S2). The greater reduction in additive genetic variation in both sexes under ML selection relative to SA is thus probably a result of the female-correlated response to directional selection on males not seen under SA selection [24]. This has important ramifications for understanding how genetic variation for sexually selected traits can be maintained (i.e. the lek paradox [52]). It is possible that traits evolving under sexual selection in one sex are in fact subject to SA selection even when they appear static in the opposite sex, which can help to explain how unexpectedly high genetic variation is maintained.

Body size is a classical polygenic trait, but our results suggest that in addition to many small effect loci there are likely a few large effect loci that underlie the body size variation. Large effect loci are a likely cause for the observed differences between the RS replicates as well as the consistency between the replicate lines under SA and ML selection. Despite the stochastic nature of genetic drift, drift should not affect replicates differently when the underlying number of loci is very high, as would be expected under infinitesimal model (i.e. the effect of drift on each locus only has a vanishingly small effect on the overall genetic variance), but can do so in the presence of large effect loci. Under selection, the stochastic effect of drift on large effect loci is likely secondary to the effect of selection itself. However, given both the persistence of $V_{A}^{a}$ for male body size even under strong directional selection, and that the selection response never plateaued in our experiment [24], our results further suggest that at least part of the genetic variance is due to many small effect loci and thus the number of underlying loci is still likely to be large (see SI Results for more details).

Maintenance of genetic variation in SA loci is explicitly predicted under sex-specific dominance [17,36]. In line with this theoretical expectation, we found that dominance variance for female body size was fully maintained under SA selection, but eroded under the directional ML selection. As the only detectable source of female-specific genetic variation, dominance variance was likely targeted by SA selection. Note however that dominance variance estimated from the pedigrees after selection have the caveat of being potentially confounded with maternal effects (*V*_ME_) (see methods). Regardless of sex, including selection lines where we found no dominance variance, *V*_ME_ was found to be zero (posterior mode = 0) in all cases. More importantly, in the ancestral population – where dominance estimates were not confounded with *V*_ME_ – we could not detect any maternal effects distinguishable from zero in either sex. In order for confounding effects of *V*_ME_ to explain our result of maintained female dominance, maternal effects specific to females would need to have arisen in the SA lines. We therefore believe that a more parsimonious explanation is that SA lines simply maintained the ancestral dominance variance and *V*_ME_ remained indistinguishable from zero.

Sex-specific dominance variance both on the X [53] and on the autosomes [4,14,16] has been considered a partial resolution to intra-locus sexual conflict, but the role of sex-specific dominance in facilitating the evolution of sexual dimorphism is not yet clear. While there is support for sex-specific dominance for sexually antagonistic traits [4,41,54] empirical tests of how SA selection affects sex-specific dominance have been lacking. Our result, which suggests a complete maintenance of female-specific dominance for body size under SA selection, showcases how such dominance can contribute to the maintenance of total genetic variance in fitness-related traits. Future work is targeted to identify the genetic basis to this variation. Given that heterozygosity is a necessary condition for dominance allelic effects to manifest themselves as dominance variation, male-limited selection may have reduced female-specific dominance by reducing allelic variation in the population, and consequently heterozygosity. The fact that the dominance signal was lost even in one of the random selection lines suggests further that SA selection is especially powerful for maintaining dominance variance.

The increase in sexual size dimorphism under male-limited selection shows, however, that female-specific dominance variance is not necessary for dimorphism to evolve. The response of dimorphism to male-limited selection can be explained by the sex-linked variance in males, particularly on the Y chromosome detected in the ancestral population, which was further demonstrated with genetic crosses [24]. The Y chromosome effectively acts as one major effect locus due to its non-recombining nature and segregating variation becomes easily fixed [55]. The effect of drift is also greater on sex-linked variation due to the lower effective population sizes of the X and Y relative to the autosomes [56]. As expected from theory, both forms of sex-specific selection (and RS) largely eroded the X and Y chromosome-linked variances limited to males.

Under the assumption that loci harbouring sex-shared and sex-specific variance are not linked, SA selection should specifically maintain sex-shared variance. The differences between SA and ML regimes should therefore be most pronounced when comparing the sex-shared variances (i.e. the between sex covariance). Albeit consistent in this direction, the difference in covariance we detected is not as large as seen in V_G_ or V_A_, suggesting that SA selection also maintained more sex-limited genetic variance compared to ML. This indicates linkage of sex-shared and sex-specific genetic variances during the artificial selection. In principle, sex-shared and sex-specific genetic variances could be connected through pleiotropy, e.g. if a growth regulating locus affects both sex-shared and sex-specific tissues and thus contributes to sex-shared as well as to sex-specific variance. Alternatively, proximity of loci contributing to each type of variance could cause linkage. Whether sex-specific variances maintained due to linkage would persist over longer evolutionary times scales under SA selection depends on their underlying cause, but if caused by selection, linkage disequilibrium is expected to eventually break down over extended periods of time.

The amount of shared genetic variance remained high during 10 generations of selection and we could not detect a significant reduction in any of the selection lines compared with the ancestral population (electronic supplementary material, table S5). Nor could we find any association between and the degree of change in sexual size dimorphism (figure 4). This further suggests that sexual size dimorphism has increased due to selection on genetic variances specific to each sex rather than a reduction in the genetic correlation between the sexes at the shared autosomal loci. Sex-specific selection is expected to erode sex-specific variance over time [57]. While the loss of for example sex-linked variance should not impact the estimates of autosomal $r_{m,f}^{a}$, it could, in fact, increase $r_{m,f}^{*}$, as the loss of sex-specific genetic variance is expected to disproportionally affect the denominator (total additive genetic variance) and not the numerator (covariance, see SI: Posterior estimate calculations) of the genetic correlation [50]. This is what we also see in our lines (electronic supplementary material, table S5).

The idea that selection for increased sexual dimorphism has the potential to maintain and even increase the $r_{m,f}$ has been discussed before [25,32,33]. But our findings are also in contrast with previous studies that have compared the degree of sexual dimorphism between different traits, and found that the $r_{m,f}$ is lower for more dimorphic traits [30]. The $r_{m,f}$ is also lower for genes with more sexually dimorphic expression [31]. The patterns across studies are not equivocal [31,33], and differences may arise from the way the $r_{m,f}$ is calculated, but also due to the fact that studies comparing the $r_{m,f}$ across genes [31] or higher organismal phenotypes [30,58] that vary in dimorphism are likely to pick up the differences among traits that arise from sex-specific genetic architectures [51]. These include for example sex differences in gene expression, sex-linkage or sex-limited duplication events [59–61], which have evolved over very long evolutionary time scales. By addressing how sexual dimorphism can evolve in a single trait from standing genetic variation our study shows instead how the $r_{m,f}$ estimates are essentially unaffected over a short time scale and are not correlated with the degree of sexual dimorphism.

## 4. Conclusion

Our results experimentally show that sexually antagonistic selection can maintain autosomal additive genetic variance longer than sex-limited directional selection, which more easily erodes variation. The shared genome is a prerequisite for genetic variation to be maintained under SA selection, but also the reason why genetic variation is lost in both sexes due to directional selection even if only one sex is under selection. Our results additionally shed new light on the historic debate over the role of dominance variation evolution [62], by showing how trait evolution under SA selection can indeed maintain sex-specific dominance variance. Understanding further how such dominance variation may evolve and contribute to sexual dimorphism is an exciting future research direction.

## 5. Methods

### (a) Study organism

The seed beetle *Callosobruchus maculatus* is a highly amenable study organism to use in tests of the quantitative genetic underpinnings of sex-specific phenotypic evolution, as it shows heritable variation in the degree of sexual size dimorphism. As a pest of legume seed pods, female *C. maculatus* oviposit directly onto seeds in which the larvae conclude their development and emerge as mature adults after 21–25 days under benign conditions (29°C, 50% RH and a 12 : 12 light : dark cycle [63]). *C. maculatus* is an aphagous capital breeder and can complete their reproductive cycle relying only on resources gathered as larvae within the seeds.

The *C. maculatus* population used to establish the selection lines for this study was created by mixing 41 isofemales lines established from wild-collected beetles. The isofemale lines were founded by randomly pairing virgin beetles collected near Lome, Togo in 2010. More detailed information about the founding populations is presented in [24].

### (b) Quantifying the effect of sex-specific selection on genetic body size variance

In this study, we experimentally tested how two different forms of sex-specific selection, sexually antagonistic (SA) selection for larger females and small males (i.e. increasing the naturally occurring SSD) and sex-limited selection for small males (ML), as well as drift due to random selection, affected different genetic variance components associated with body size evolution during 10 generations of artificial selection. To do this, we compared the lines after selection to each other, to random selection, and to the ancestral population prior to the selection period. For this, we capitalized on data on the ancestral population presented in [24], where we described the genetic variance in body size in each sex in the ancestral population and show that different artificial selection regimes yield different phenotypic responses.

#### (i) Pedigree design and artificial selection

Here we provide an overview of the quantitative genetic and artificial selection methods applied to the ancestral population. Further details can be found in [24]. An extensive quantitative genetics experiment including 8022 individuals of the ancestral population was carried out prior to the present study, where we characterized the genetic variances of body size in a *C. maculatus* population before selection (i.e. in the ancestral population) using a pedigree design adapted from [61] specifically useful for quantifying X-linked and dominance variances. We then used the offspring of this quantitative genetics experiment as the basis to start 10 generations of artificial selection under different selection regimes, where we applied sexually antagonistic selection (SA), male-limited directional selection (ML) or random selection (RS), respectively. In each selection regime we selected the top 8 families out of 56 families based on the following criteria: 8 families with the highest degree of SSD (measured as the ratio between average female and male body weight) for the SA lines, 8 families with the smallest average male body weight (i.e. no selection on females) for the ML lines, and 8 randomly selected families for the drift lines. Within each selection regime, selected families were crossed fully factorially, but excluding full sib crossings, resulting in 56 new families in the next generation. Each selection regime was replicated twice (hereafter, replicates A and B). After the last generation of selection, the top 8 families, chosen using the same criteria as before, were pooled within each selection regime and continued as a panmictic population, building the basis for the follow-up quantitative genetics experiment presented in this study where we tested the genetic variances for body size in each population after selection.

#### (ii) Pedigree design for this study

To reach comparable sample sizes to the quantitative genetic pedigree analyses conducted for ancestral generation we split the line replicates into two blocks. The present quantitative genetics experiments for all three selection regimes of replicate A were started in parallel after one generation of panmictic mating after selection. One generation later (i.e. after two generations of panmictic mating), the breeding design for the selection regimes in replicate B were started as well. We performed the quantitative genetics experiment for each selection regime and replicate as described below.

Similar to the quantitative genetics experiment using in the ancestral population, we used a pedigree design adapted from [61] spanning 3 generations, GP, P and F1, in order to partition dominance, additive sex-linked and additive autosomal variances for each sex and the cross-sex covariance. Note that, unlike in the ancestral pedigree used in [24], in the present study (due to logistic constrains) we had to create slightly reduced pedigrees by omitting the double first cousins. While these types of relatives are important for partitioning out potential confounding of maternal effects ($V_{\mathrm{ME}}$) on $V_{D}$ as well as $V_{A}^{X}$ estimates, which we now cannot do, we note that in the ancestral dataset we could demonstrate that there is no effect of $V_{\mathrm{ME}}$ on body size in either sex (i.e. the posterior mode of $V_{\mathrm{ME}}$ is indistinguishable from zero in both sexes) (electronic supplementary material, table S1). Hence, we believe that we can accurately partition dominance and X-linked variances. Note also that any confounding maternal effects in the present pedigrees would need to be female-specific and to have occurred only after selection, in order to account for the results we observe (i.e. maintenance of ancestral levels of female-specific dominance under SA selection). All adults were collected as virgins by isolating the host seeds 5 days prior to emergence of the adult beetles. To ensure the virgin status of the beetles and to rule out potential larval resource competition, we only isolated beans with one egg laid on them and thus only one growing larva inside. Additionally, to keep the conditions consistent across all experiments, we measured all individuals within 24 h of their emergence using a set of three micro scales and kept the populations density controlled starting two generations—with the great-great-grandparents (GGGP)—before quantifying the first experimental individuals. All crosses were done by pairing a mature virgin female with a mature virgin male in a 1.5 ml Eppendorf for one hour, the only exception to this was in the parental crossing scheme (see below) where we crossed males to multiple females (i.e. males were no longer virgins after the first mating). Thereafter, we transferred the mated females into individual Petri dishes prepared with 18 g *V. radiata* beans for 24 h of oviposition. Both crossing and oviposition were done under benign conditions in a climate chamber.

To initiate the quantitative genetic experiments, randomly collected adult *C. maculatus* (GGGP) from the panmictic population were transferred to a new set of *V. radiata* beans for oviposition. As in the ancestral population, adult beetles were removed 48 h later to unify the age of the following great-grandparental generation (GGP) and to control and equalize density across all regimes. GGP individuals were collected as virgins and randomly crossed to create 120 GP families. Note that beyond controlling for age and density, the panmictic mating as well as the random paring of GGGP and GGP generations serves two crucial purposes in our experiment: (i) minimizing potential relatedness structure which would violate assumptions of the animal model and (ii) removing potential build-up linkage disequilibrium that can bias additive genetic variance to be overestimated [64].

GP generation: From each GP family we selected one individual at random but balanced for sex, yielding 120 unrelated beetles, 60 females and 60 males, respectively. Males and females were randomly crossed to give rise to 60 P families.

P generation: For each parental family we randomly selected 1 male and 4 females. Each male was sequentially crossed to 4 randomly selected females of different parental families, giving rise to 4 half-sib families.

In total we measured approximately 2000 beetles in each regime and replicate (detailed sample sizes can be found in the respective electronic supplementary material, tables).

### (c) Quantitative genetics analysis

To detect potential changes in genetic variances due to the response to artificial selection we analysed the pedigrees of each artificial selection and drift line with the animal model (mixed effect model) in the software R, v. 3.4.0 [65]. We used the same animal model framework as described in [24] for the ancestral population, where we modelled body size as a bivariate, normally distributed trait in a GLMM with Gaussian errors and an identity link function using the R package MCMCglmm [66]. Using this model allowed us to partition the observed phenotypic body size variation into: autosomal additive genetic variance ($V_{A}^{a}$), cross-sex autosomal additive covariance (COV) and the associated intersex genetic correlation ($r_{m,f}$); sex-specific X-linked additive genetic variances ($V_{A}^{X}$) and Y-linked additive variance ($V_{A}^{Y}$); dominance variances ($V_{D}$); and maternal environment ($V_{ME}$). In this manuscript, we present our results using parameter expanded, uninformative priors but we also explored other prior conditions in order to validate the robustness of our model. Additive, dominance and X-linked (co)-variance relationships were calculated using the R package NADIV [67]. In the animal model, the estimation of the covariance for dominance and additive X-linked variance were fixed to zero *a priori*. The R code of our models is provided in the SI. In addition to the Bayesian framework we also used a likelihood based method to verify our findings with a different method and explore model comparison using the software ASReml R 4.0. All calculations of associated statistics such as sex specific heritabilities and $r_{m,f}$ are in the supplementary information.

We created posterior differences between marginal posterior distributions of the replicate lines to compare the genetic variance component estimates against the ancestral population, as well as within and across selection regimes. Additionally, we compared the male-female covariance estimates between the replicates of the SA and ML regimes. The posterior difference itself is a posterior distribution and statistical inference can be drawn similarly as for all posterior distributions (i.e. primarily via 95% CI). After verifying that there were no significant differences between the replicate lines within the SA or the ML selection regimes, we estimated the genetic variance components for each selection regime (i.e. including both A & B replicate lines in the same model) to compare the effects of SA versus ML selection on genetic variance by creating a posterior difference distribution between the regimes. Additionally, we also present individually the posterior difference distribution comparison across all SA and ML replicate lines to demonstrate the consistency of our findings (electronic supplementary material, figure S7).

## Data Availability

All data generated and analysed in this study are published on the Dryad repository [68]. Data for the ancestral population are published on the Dryad repository [69]. Supplementary data are provided in the electronic supplementary material [70].
